# Effect of Licochalcone A on Growth and Properties of *Streptococcus suis*

**DOI:** 10.1371/journal.pone.0067728

**Published:** 2013-07-23

**Authors:** Huaijie Hao, Wenjia Hui, Peng Liu, Qingyu Lv, Xiaotao Zeng, Hua jiang, Yanzi Wang, Xin Zheng, Yuling Zheng, Jianchun Li, Xuyu Zhou, Yongqiang Jiang

**Affiliations:** 1 State Key Laboratory of Pathogen and Biosecurity, Institute of Microbiology and Epidemiology, Academy of Military Medical Sciences, Beijing, China; 2 CAS Key Laboratory of Pathogenic Microbiology and Immunology, Institute of Microbiology, Chinese Academy of Sciences, Beijing, China; 3 Department of Traditional Chinese Medicine Pharmacology, Shenyang Pharmaceutical University, Shenyang, China; 4 Department of Pharmacy, Jiangsu Provincial Xuzhou Pharmaceutical Vocational College, Xuzhou, China; 5 Department of Pharmacology, Shenyang Pharmaceutical University, Shenyang, China; University of Kansas Medical Center, United States of America

## Abstract

*Streptococcus suis* (*S.suis*) is an important emerging worldwide pig pathogen and zoonotic agent with rapid evolution of virulence and drug resistance. In this study, we wanted to investigate the effect of licochalcone A on growth and properties of *Streptococcus suis*. The antimicrobial activity of licochalcone A was tested by growth inhibition assay and the minimal inhibitory concentrations (MICs) also were determined. The effect of licochalcone A on *S.suis* biofilm formation was characterized by crystal violet staining. The effect of licochalcone A on suilysin secretion was evaluated by titration of hemolytic activity. To understand the antimicrobial effect, gene expression profile of *S.suis* treated by licochalcone A was analyzed by DNA microarray. Our results demonstrated that licochalcone A showed antimicrobial activity on *S.suis* with MICs of 4 µg/ml for *S.suis* serotype 2 strains and 8 µg/ml for *S.suis* serotype 7 strains. Biofilm formation was inhibited by 30–40% in the presence of licochalcone A (3 µg/ml) and suilysin secretion was also significantly inhibited in the presence of licochalcone A (1.5 µg/ml). The gene expression profile of *S.suis* in the presence of licochalcone A showed that 132 genes were differentially regulated, and we analyzed the regulated genes in the aspect of the bacterial cell cycle control. Among the deregulated genes, the genes responsible for the mass doubling was increased expression, but the genes responsible for DNA replication and cell division were inhibited the expression. So, we think the regulation of the cell cycle genes might provide a mechanistic understanding of licochalcone A mediated antimicrobial effect against *S.suis*.

## Introduction

*Streptococcus suis* (*S.suis*) is an important emerging worldwide pig pathogen and zoonotic agent causing severe meningitis, pneumonia, and sepsis in pigs and also meningitis and Streptococci toxin-shock-like syndrome (STSLS) in humans [1], [2]. Since the first human infection report in Denmark in 1968, infections in humans were considered sporadic infections in people working with pigs or pork-derived products [3]. However, a large outbreak of *S.suis* serotype 2 infection that emerged in Sichuan Province, China in 2005 and resulted in 215 cases and 38 deaths among humans, has changed the perspective of the threat posed by this pathogen to human health [4].

For *S.suis* infection in human, intravenous penicillin G has been used to successfully treat most cases. However, penicillin-resistant strains have been isolated in 6–28% of piglets. On the other hand, the widespread use of antibiotics such as tetracycline in swine feed have been demonstrated to provide the selective pressure for rapid evolution of virulence and drug resistance in *S.suis* [5], [6]. Up to now, *S.suis* vaccines for humans do not exist so far and there are also no effective vaccines available even for swine.

Licochalcone A is one of the many flavonoids present in the Chinese licorice root, which is used in traditional Chinese medicine. The structure of licochalcone A was first reported in 1975, but no biological activity was described [7]. Later studies have revealed that licochalcone A exhibits antimicrobial, antioxidant and anti-inflammatory activities [8]–[11]. In this study, we investigated the effect and possible mechanism of licochalcone A on growth and properties of *S.suis*.

## Results

### Growth of *Streptococcus suis* in the presence of licochalcone A

**Figure 1:**
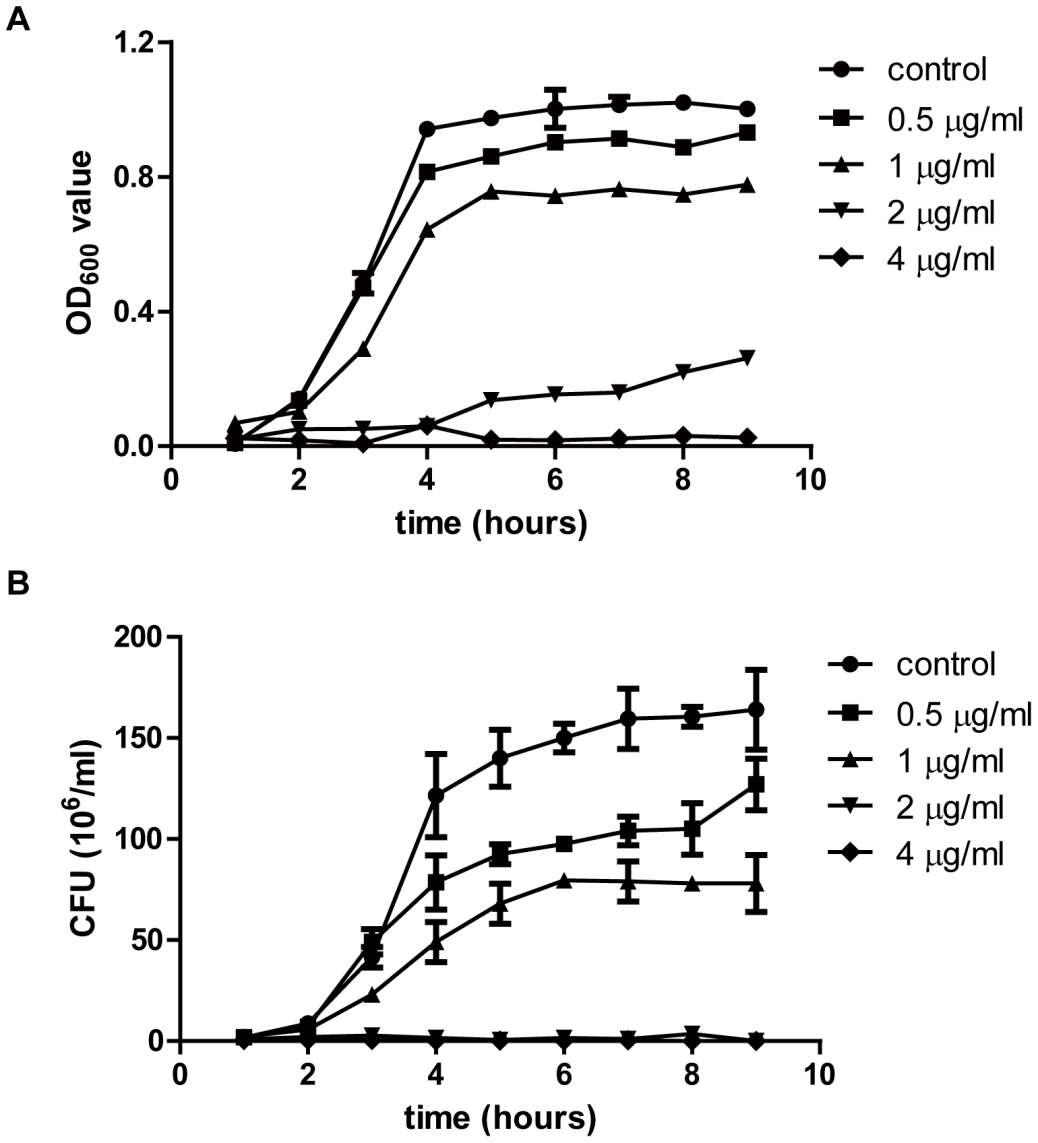
Effects of licochalcone A on the in vitro growth of *S.suis* strain 05ZYH33. (A) the absorbance of bacteria at 600 nm at different time. (B) the viable bacteria number at different time. CFU: colony forming unit.

The growth curves of *S.suis* strain 05ZYH33 is shown in Figure 1. The data of bacteria density and colony forming unit (CFU) showed that the growth of bacteria was inhibited in a licochalcone A concentration-dependent manner. At 2 µg/ml, licochalcone A could show the bacteriostatic effect on the growth of *S.suis* strain 05ZYH33. At 4 µg/ml, licochalcone A completely inhibited bacteria growth.

To further evaluate the activity of licochalcone A against *S.suis*, the MICs of different *S.suis* strains were determined. As shown in Table 1, licochalcone A was effective for all tested *S.suis* strains with MIC of 4 µg/ml for *S.suis* serotype 2 strains and 8 µg/ml for *S.suis* serotype 7 strains.

**Table 1:** Antimicrobial activity of licochalcone A against *Streptococcus suis*.

Strain	Description^a^	Origin^b^	MIC (µg/ml)
***S.suis*** ** 05ZYH33**	Serotype 2, MRP^+^EF^+^SLY^+^89K^+^	Sichuan, China HP, 2005	4
***S.suis*** ** 5**	Serotype 2, MRP^+^EF^+^SLY^+^89K^+^	Sichuan, China DP, 2005	4
***S.suis*** ** 98012**	Serotype 2, MRP^+^EF^+^SLY^+^89K^+^	Jiangsu, China HP, 1998	4
***S.suis*** ** 98013**	Serotype 2, MRP^+^EF^+^SLY^+^89K^+^	Jiangsu, China HP, 1998	4
***S.suis*** ** 2005001**	Serotype 2, MRP^+^EF^+^SLY^+^89K^+^	Sichuan, China HP, 2005	4
***S.suis*** ** 2005002**	Serotype 2, MRP^+^EF^+^SLY^+^89K^+^	Sichuan, China HP, 2005	4
***S.suis*** ** sun**	Serotype 2, MRP^+^EF^+^SLY^+^89K^+^	Jiangsu, China HP, 2006	4
***S.suis*** ** 606**	Serotype 2, MRP^+^EF^+^SLY^+^89K^−^	China DP, 1980	4
***S.suis*** ** 1940**	Serotype 2, MRP^+^EF^+^SLY^+^89K^−^	China DP, 1980	4
***S.suis*** ** 1941**	Serotype 2, MRP^+^EF^+^SLY^+^89K^−^	China DP, 1980	4
***S.suis*** ** NJ**	Serotype 2, MRP^+^EF^+^SLY^+^89K^−^	Jiangsu, China DP	4
***S.suis*** ** S735**	Serotype 2, MRP^+^EF^+^SLY^+^89K^−^	Netherlands DP	4
***S.suis*** ** 4005**	Serotype 2, MRP^+^EF^+^SLY^+^89K^−^	Netherlands DP	4
***S.suis*** ** F8-2**	Serotype 7	Hebei, China HPL	8
***S.suis*** ** B11**	Serotype 7	Hebei, China HPL	8
***S.suis*** ** M20-1**	Serotype 7	Hebei, China HPL	8
***S.suis*** ** 68-1**	Serotype 7	Hebei, China HPL	8
***S.suis*** ** 5B2**	Serotype 7	Jilin, China HPL	8

a 89K, 89 K pathogenicity islands (PAI).

b HP, human patients; DP, diseased piglets; HPL, healthy piglets.

### Licochalcone A inhibits the biofilm formation of *Streptococcus suis*

Bacterial biofilms are formed when unicellular organisms come together to form a community that is attached to a solid surface and encased in an exopolysaccharide matrix. It has been suggested that this matrix, among other functions, prevents the access of antibiotics to the bacterial cells embedded in the community. When bacteria exist in a biofilm, they can become 10–100 times more resistant to the effects of antimicrobial agents [12], [13].

**Figure 2:**
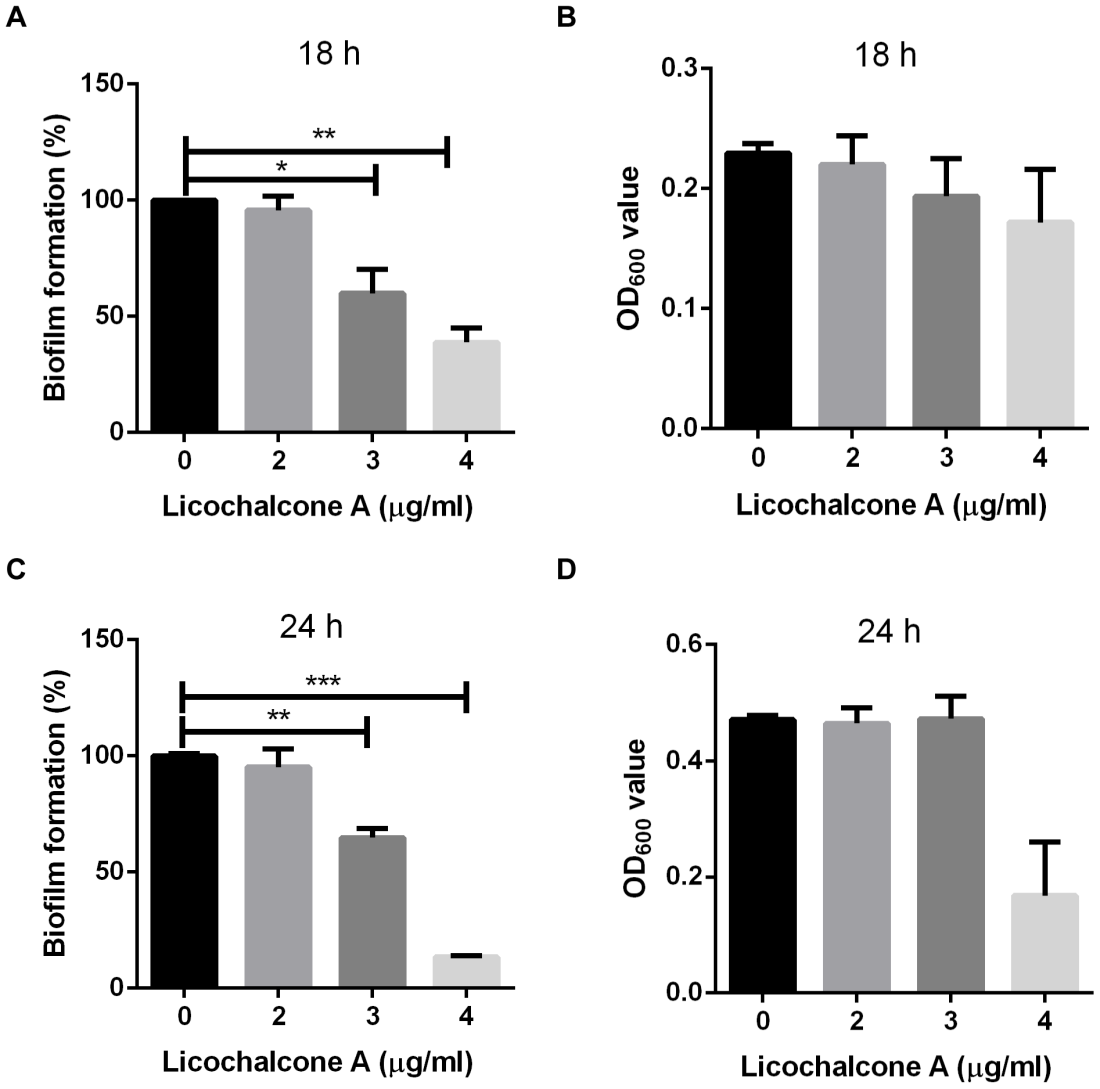
Effect of licochalcone A on biofilm formation by *S.suis* serotype 2 strain 05ZYH33 determined by the microtiter plate assay. *S.suis* was cultured in THB medium supplemented with 5 mg/ml human fibrinogen for 18 h (A) or 24 h (C) and biofilm formation was determined in the presence of 0, 2, 3 and 4 µg/ml licochalcone A, respectively. A value of 100% was given to the biofilm formed in the absence of licochalcone A. Assays were performed in triplicate, and the means ± standard deviations of two independent experiments are indicated. The total cell density at 18 h (B) or 24 h (D) also were measured spectrophotometrically (OD600 nm).

*S.suis* is capable of forming a dense biofilm especially in the presence of fibrinogen [14]. In this study, we evaluated the effect of licochalcone A on biofilm formation. Biofilm was stained with crystal violet and the absorbance value at 550 nm was determined the biofilm formation. As shown in Figure 2A and 2C, biofilm formation was inhibited with the increasing the concentration of licochalcone A, and concentration of licochalcone A at 3 µg/ml still had a significant inhibitory effect on biofilm formation. To investigate if the inhibition of biofilm formation in the presence of licochalcone A was due to the retardation of growth, the total cell density (biofilm and planktonic cells) was determined at 18 h or 24 h. As shown in Figure 2B and 2D, it had no significantly influence on the total cell density of *S.suis* strain 05ZYH33 at the concentration of 3 µg/ml licochalcone A. The above concentration (3 µg/ml) is lower than the MIC values, suggesting a true specific anti-biofilm effect for licochalcone A on *S.suis*.

### Licochalcone A inhibits the release of suilysin

Suilysin is the hemolysin of *S.suis* serotype 2 encoded by *sly* (05SSU1403), and is a member of the thiol-activated pore-forming toxin family. Suilysin is actively involved in *S.suis* infection and host response. During interaction with human cells, suilysin was one component that up-regulated surface molecules of human monocytes [15]. The presence of suilysin could enhance epithelial invasion and cell lysis by virulent strains of *S.suis* [16]. A further study speculated that suilysin was involved in adherence and cell injury rather than in direct cellular invasion. A retrospective study correlated the presence of the suilysin gene and expression of MRP and EF with high virulence in *S.suis* serotype 2 isolates [17].

**Figure 3:**
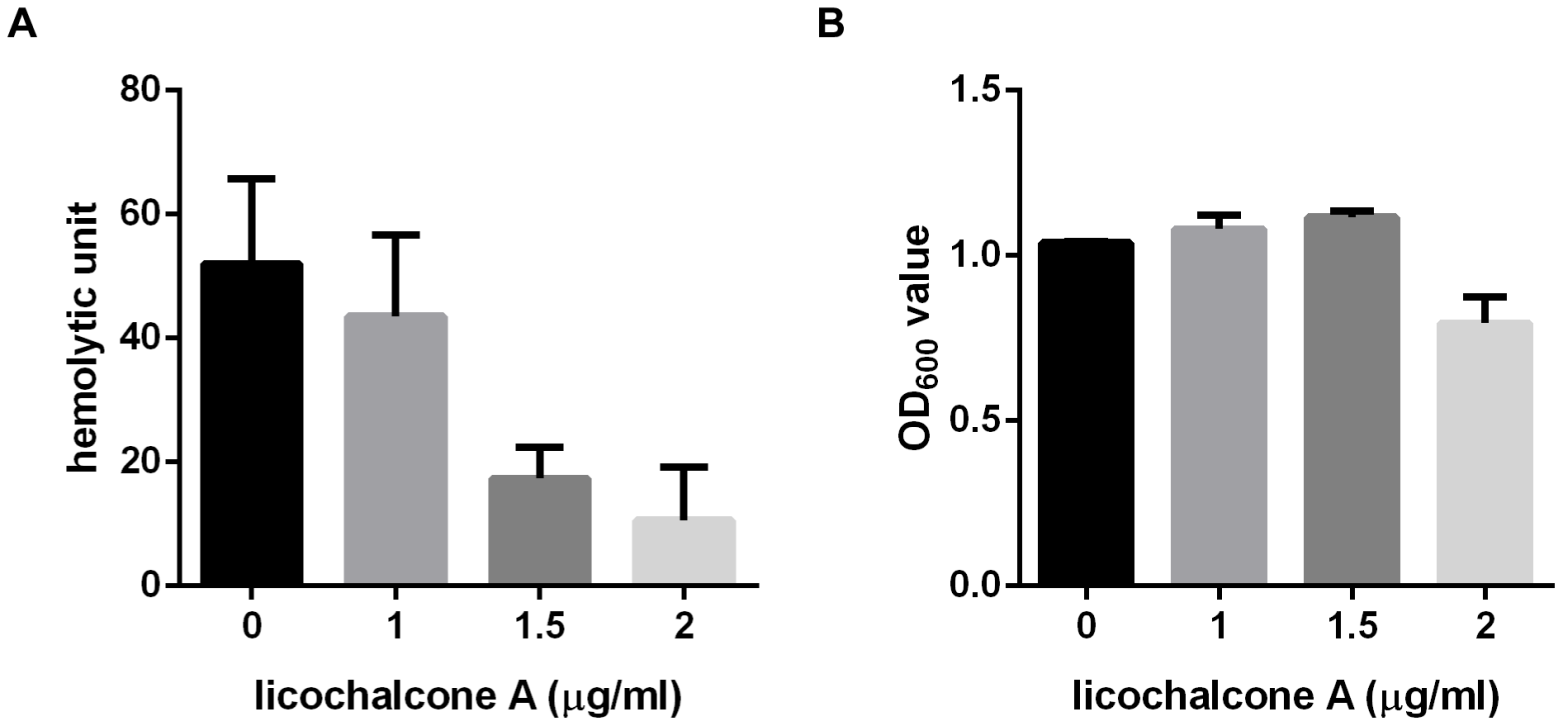
Effect of licochalcone A on suilysin secretion of *S.suis* strain 05ZYH33 determined by hemolytic activity. A. Culture supernatants of *S.suis* 05ZYH33 treated by different concentration of licochalcone A were collected and tested the hemolytic activity as described by materials and methods. One hemolytic unit is defined as the reciprocal of the suilysin titre, which was calculated as the highest dilution of the supernatant which caused at least 50% hemolysis. B. The absorbance at 600 nm was recorded to determine the changes in growth and culture density.

Effect of licochalcone A on suilysin secretion was tested by titration of hemolytic activity. *S.suis* strain 05ZYH33 was cultured at 37°C for 8 h in the presence of different concentration of licochalcone A. The culture supernatants were collected by centrifugation and tested the hemolytic activity as described in materials and methods. The reciprocal of the highest dilution of a given cell-free supernatant that exhibited at least 50% of RBC lysis was taken as the titre, in hemolytic units (HU), of the suilysin in that sample. As shown in Figure 3A, hemolytic activity of suilysin was significantly decreased when *S.suis* strain was cultured in the presence of licochalcone A (1.5 µg/ml). At the same time, we measured the bacteria density to investigate if the decrease of suilysin release was due to simply fewer cells present in samples treated with licochalcone A. As shown in Figure 3B, there were nearly same bacteria cells treated with 1.5 µg/ml licochalcone A compared with that of untreated group. These results indicated that licochalcone A could inhibit the secretion of suilysin in *S.suis* in the presence of 1.5 µg/ml licochalcone A.

The mechanism of suilysin regulation is not well clear, and environmental changes such as pH shift, nutrient exhaustion, cell confluence, or other stressful factors may play a role. A transposon mutagenesis study had demonstrated that manN, putative mannose-specific phosphotransferase system component IID encoded by 05SSU1780, repressed the expression of suilysin [18]. In this study, after treated by licochalcone A, we also found that the expression of manN was upregulated in correlation with the down-regulated expression of suilysin in *S.suis* strain 05ZYH33 (Table 2). These results indicated that manN might be a gene targeted by licochalcone A, and the effect mechanism of licochalcone A on suilysin expression by *S.suis* was similar to that of licochalcone A on alpha-toxin expression by Methicillin-sensitive *Staphylococcus aureus* (MSSA) and Methicillin-resistant *Staphylococcus aureus* (MRSA), namely targeting the regulation genes [19], [20].

**Table 2:** List of significant regulated genes of *S.suis* in the presence of licochalcone A.

Gene ID	Gene name	COG	Fold change*	Annotation
**Energy production and conversion**				
**05SSU1205**	-	C	2.23	Aconitase A
**05SSU0280**	-	C	−11.97	NAD-dependent aldehyde dehydrogenase
**Cell division and chromosome partitioning**				
**05SSU0417**	gpsB	D	−2.06	Cell division initiation protein
**05SSU0479**	-	D	−2.91	Actin-like ATPase involved in cell division
**Amino acid transport and metabolism**				
**05SSU0252**	-	E	3.22	NAD(P)H-dependent glutamate dehydrogenase
**05SSU0791**	-	E	2.05	Carbamoylphosphate synthase small subunit
**05SSU1026**	-	E	2.12	ABC-type amino acid transport/signal transduction system, periplasmic component/domain
**05SSU1027**	-	E	2.91	ABC-type amino acid transport/signal transduction system, periplasmic component/domain
**05SSU1028**	-	E	2.85	ABC-type amino acid transport/signal transduction system, periplasmic component/domain
**05SSU1029**	-	E	3.21	ABC-type polar amino acid transport system, ATPase component
**05SSU1361**	pyrC	E	3.33	ABC-type polar amino acid transport system, ATPase component
**05SSU1362**	-	E	2.22	ABC transporter substrate-binding protein - glutamine transport/Major cell binding factor precursor
**05SSU1363**	-	E	4.22	amino acid ABC transporter, permease protein
**05SSU1365**	-	E	3.89	ABC-type amino acid transport system, permease component
**05SSU1546**	-	E	2.13	ABC-type branched-chain amino acid transport system, permease component
**05SSU2067**	-	E	3.05	putative amino acid ABC transporter, ATP-binding protein
**05SSU2068**	-	E	2.81	ABC-type amino acid transport system, permease component
**05SSU1709**	pepC	E	−2.65	cysteine aminopeptidase C
**05SSU0345**	-	E	−2.18	L-asparaginase/archaeal Glu-tRNAGln amidotransferase subunit D
**Nucleotide transport and metabolism**				
**05SSU0091**	adk	F	2.98	Adenylate kinase and related kinases
**05SSU1207**	-	F	2.12	Ribonucleotide reductase, alpha subunit
**05SSU1209**	nrdF	F	2.27	Ribonucleotide reductase, beta subunit
**05SSU1935**	-	F	2.31	Xanthine/uracil permease
**05SSU2116**	-	F	2.50	5′-nucleotidase/2′,3′-cyclic phosphodiesterase and related esterases
**05SSU2118**	-	F	2.81	Oxygen-sensitive ribonucleoside-triphosphate reductase
**05SSU0357**	-	F	−2.37	deoxyguanosinetriphosphate triphosphohydrolase-related protein
**05SSU0491**	-	F	−3.01	putative hydrolase (MutT family)
**05SSU0661**	-	F	−2.63	Thymidylate kinase
**05SSU0846**	-	F	−2.07	Thymidine kinase
**05SSU1971**	-	F	−2.00	unknown protein
**05SSU1065**	-	F	−2.00	Phosphoribosylpyrophosphate synthetase
**Carbohydrate transport and metabolism**				
**05SSU1779**	manM	G	2.29	mannose-specific PTS IIC
**05SSU1780**	manN	G	2.68	mannose-specific PTS IID
**05SSU1317**	-	G	−2.24	hypothetical protein
**Coenzyme transport and metabolism**				
**05SSU0689**	-	H	−2.35	Phosphopantothenoylcysteine synthetase/decarboxylase
**05SSU0835**	-	H	−2.17	Dihydrofolate reductase
**05SSU1441**	hemN	H	−2.09	coproporphyrinogen III oxidase
**05SSU1972**	-	H	−2.21	Nicotinamide mononucleotide transporter
**05SSU1973**	-	H	−2.07	unknown protein
**Lipid metabolism**				
**05SSU1796**	-	I	2.94	Acetyl-CoA carboxylase alpha subunit
**05SSU1797**	-	I	2.87	Acetyl-CoA carboxylase beta subunit
**05SSU1798**	-	I	2.86	Acetyl-CoA carboxylase beta subunit
**05SSU1799**	accC	I	2.79	Biotin carboxylase
**05SSU1800**	-	I	2.47	3-hydroxymyristoyl/3-hydroxydecanoyl-(acyl carrier protein) dehydratase
**05SSU1804**	-	I	2.16	(acyl-carrier-protein) S-malonyltransferase
**Inorganic ion transport and metabolism**				
**05SSU0647**	-	P	2.20	ABC-type Fe3+-siderophore transport system, permease component
**05SSU1768**	-	P	2.65	ABC-type metal ion transport system, permease component
**05SSU1769**	-	P	2.44	ABC-type metal ion transport system, ATPase component
**05SSU0310**	Fur/Zur	P	−3.10	Fe2+/Zn2+ uptake regulation protein
**05SSU1689**	-	P	−2.43	ABC-type molybdenum transport system, ATPase component/photorepair protein PhrA
**05SSU1759**	-	P	−2.23	potassium uptake protein, Trk family
**05SSU1760**	-	P	−2.09	K+ transport system, NAD-binding component
**05SSU2032**	-	P	−2.30	conserved hypothetical protein
**Translation, ribosomal structure and biogenesis**				
**05SSU0071**	rplC	J	2.76	Ribosomal protein L3
**05SSU0072**	rplD	J	2.82	Ribosomal protein L4
**05SSU0073**	rplW	J	3.12	Ribosomal protein L23
**05SSU0074**	rplB	J	3.22	Ribosomal protein L2
**05SSU0075**	rpsS	J	3.24	SSU ribosomal protein S19P
**05SSU0076**	rplV	J	3.65	Ribosomal protein L22
**05SSU0077**	rpsC	J	3.73	ribosomal protein S3
**05SSU0078**	rplP	J	3.83	50S ribosomal protein L16
**05SSU0079**	-	J	3.91	50s ribosomal protein L29
**05SSU0081**	rplN	J	4.03	Ribosomal protein L14
**05SSU0082**	rplX	J	3.21	Ribosomal protein L24
**05SSU0083**	rplE	J	3.84	Ribosomal protein L5
**05SSU0085**	rplF	J	3.95	Ribosomal protein L6P/L9E
**05SSU0086**	rplR	J	3.39	Ribosomal protein L18
**05SSU0087**	rpsE	J	4.52	Ribosomal protein S5
**05SSU0089**	-	J	4.62	Ribosomal protein L15
**05SSU0151**	-	J	2.63	Ribosomal protein S7
**05SSU0152**	-	J	2.51	Translation elongation factor (GTPases)
**05SSU0983**	rplL	J	3.11	Ribosomal protein L7/L12
**05SSU0984**	-	J	2.81	Ribosomal protein L10
**05SSU1268**	rplT	J	2.84	Ribosomal protein L20
**05SSU1269**	rpmI	J	2.32	50S ribosomal protein L35
**05SSU1979**	tsf	J	2.33	Translation elongation factor Ts
**05SSU1980**	rpsB	J	2.39	30S ribosomal protein S2
**05SSU2156**	rpsD	J	2.62	30S ribosomal protein S4
**Transcription**				
**05SSU0095**	-	K	2.52	DNA-directed RNA polymerase, alpha subunit/40 kD subunit
**05SSU0122**	-	K	3.30	DNA-directed RNA polymerase, beta' subunit/160 kD subunit
**05SSU0395**	-	K	−2.01	Transcriptional regulator
**05SSU0608**	-	K	−2.10	Transcriptional regulator
**05SSU1012**	-	K	−2.09	Transcriptional regulator
**05SSU2056**	-	K	−2.06	Transcriptional antiterminator
**DNA replication, recombination and repair**				
**05SSU1833**	-	L	2.22	putative single-stranded DNA-binding protein
**05SSU1047**	-	L	−2.99	Integrase
**Cell envelope biogenesis, outer membrane**				
**05SSU2099**	-	M	4.42	hypothetical protein
**05SSU2100**	-	M	6.66	Unknown protein
**05SSU2103**	-	M	4.47	LPXTG-motif cell wall anchor domain protein
**05SSU2104**	-	M	4.49	hypothetical protein
**05SSU0745**	-	M	−2.06	D-alanyl-D-alanine carboxypeptidase
**05SSU1370**	-	M	−2.24	Glycosyltransferase
**Posttranslational modification, protein turnover, chaperones**				
**05SSU0149**	-	O	2.04	GroEL
**05SSU0299**	-	O	2.45	Molecular chaperone GrpE (heat shock protein)
**05SSU0300**	-	O	2.64	Molecular chaperone
**05SSU0389**	-	O	2.50	ATPases with chaperone activity, ATP-binding subunit
**05SSU0390**	-	O	2.70	ATPases with chaperone activity, ATP-binding subunit
**05SSU1344**	-	O	−2.09	Peptidyl-prolyl cis-trans isomerase (rotamase) - cyclophilin family
**General function prediction only**				
**05SSU1257**		R	2.59	ABC transporter permease protein
**05SSU1894**	-	R	2.06	Predicted metal-sulfur cluster biosynthetic enzyme
**05SSU2115**	-	R	2.04	Predicted acetyltransferase
**05SSU0228**	-	R	−2.13	Predicted dehydrogenase and related proteins
**05SSU0362**	-	R	−2.05	Permease of the drug/metabolite transporter (DMT) superfamily
**05SSU0279**	-	R	−4.42	Zn-dependent alcohol dehydrogenase
**05SSU0346**	-	R	−2.03	Predicted hydrolase of the HAD superfamily
**05SSU0365**	-	R	−2.52	Predicted hydrolase of the HAD superfamily
**05SSU1264**	-	R	−2.04	Predicted SAM-dependent methyltransferase
**05SSU1399**	-	R	−2.24	hypothetical protein
**05SSU1496**	-	R	−2.00	Predicted permease
**05SSU1668**	-	R	−2.08	Hemolysins and related proteins containing CBS domains
**Function unknown**				
**05SSU1690**	-	S	−2.92	SAM-dependent methyltransferase
**05SSU1707**	-	S	−2.07	Uncharacterized protein conserved in bacteria
**05SSU0875**	-	S	−2.33	Uncharacterized protein conserved in bacteria
**Intracellular trafficking, secretion and vesicular transport**				
**05SSU0090**	-	U	4.38	Preprotein translocase subunit SecY
**05SSU1965**	-	U	−2.22	unknown protein
**Defense mechanisms**				
**05SSU1450**	-	V	−2.20	Type I restriction enzyme EcoKI specificity protein (S protein)
**Not in COGs**				
**05SSU0473**	-		2.47	Ribonucleases G and E
**05SSU0474**	-		2.65	Autotransporter adhesin
**05SSU1792**	-		2.06	Unknown protein
**05SSU1793**	-		2.09	hypothetical protein
**05SSU1895**	-		2.08	Uncharacterized conserved protein
**05SSU2101**	-		7.61	Unknown protein
**05SSU0139**	-		−2.07	hypothetical protein
**05SSU0227**	-		−2.01	Predicted dehydrogenase and related proteins
**05SSU0870**	-		−3.09	hypothetical protein
**05SSU1082**	-		−2.75	hypothetical protein
**05SSU1274**	-		−2.11	hypothetical protein
**05SSU1403**	sly		−3.25	Hemolysin, also named suilysin
**05SSU1503**	-		−2.57	putative enolase
**05SSU1553**	-		−2.35	Uracil phosphoribosyltransferase

* Positive number represents fold change of upregulated gene, and negative number represents fold change of downregulated gene at the condition of licochalcone A treatment versus untreated reference condition.

### Gene expression profile of *Streptococcus suis* treated by subinhibitory concentration of licochalcone A

**Figure 4:**
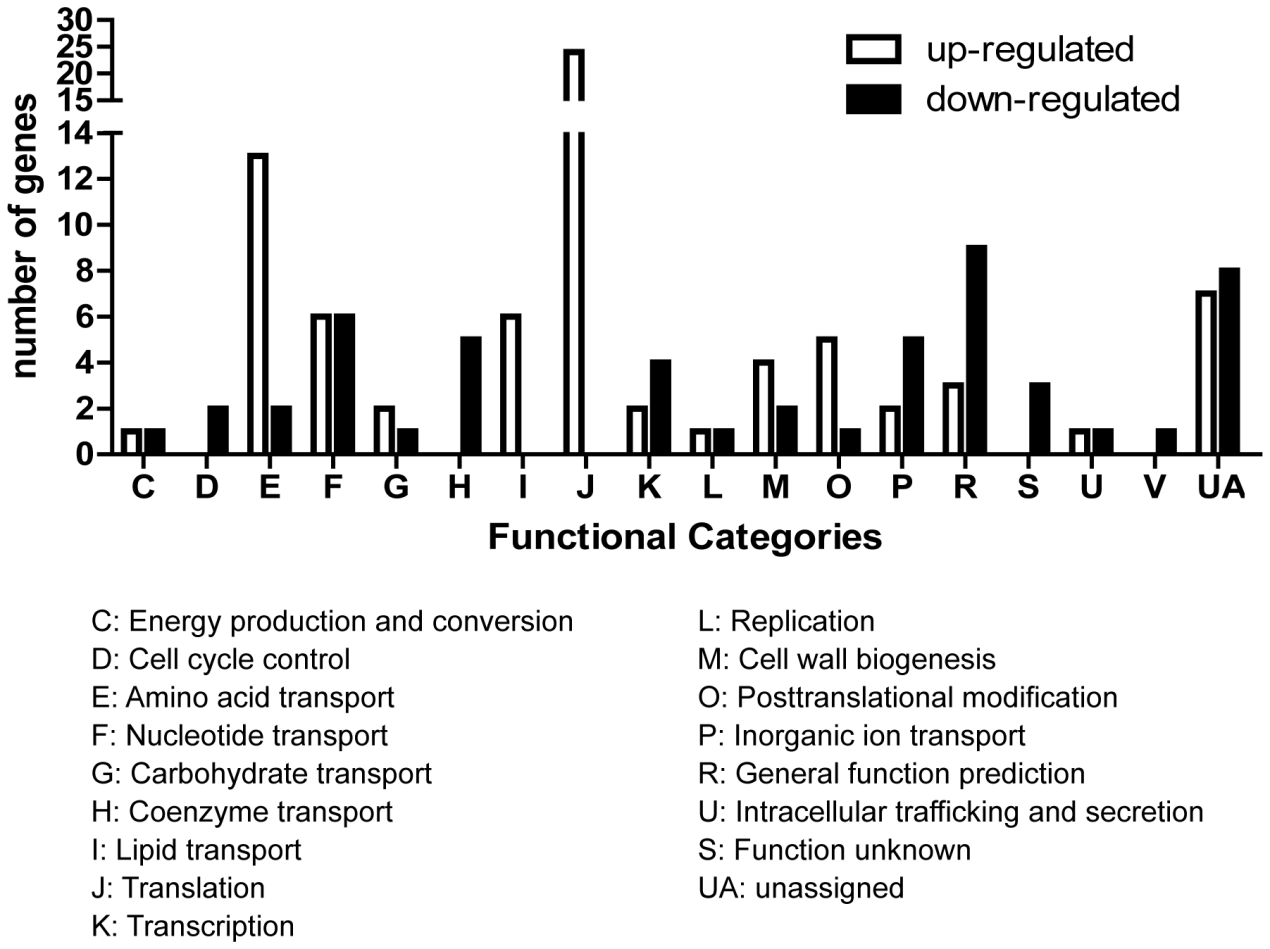
Differentially regulated genes (more than twofold changes) grouped by functional classification according to *Streptococcus suis* strain 05ZYH33 genome annotation. The differentially regulated genes on the chromosome were divided into 18 categories. The number of genes up-regulated and down-regulated for each functional group was represented.

**Figure 5:**
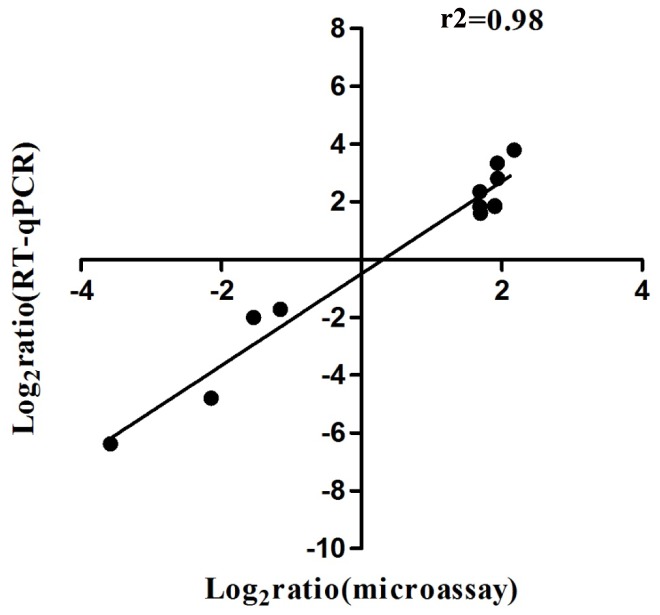
Comparison of microarray and RT-PCR data. The relative transcriptional levels of 11 genes were determined by microarray and real-time RT-PCR. The fold changes in gene transcription in response to subinhibitory concentration of licochalcone A were logarithm-transformed in base 2. The real-time RT-PCR log2 values were plotted against the microarray data log2 values.

The effect of licochalcone A on growth and properties of *S.suis* indicated its therapeutic potential for *S.suis* infection. To further explore the molecular mechanism of the effect, we compared the gene expression profile of *S.suis* serotype 2 strain 05ZYH33 that was cultured in the presence of subinhibitory concentration of licochalcone A and in the THB medium only. Of the 1930 genes whose mRNA expression was detected by microarray, 132 genes were differentially regulated upon licochalcone A treatment, including 78 genes (59%) up-regulated and 54 genes (41%) down-regulated. The 132 regulated genes could be assigned into 18 function categories (COG) based on the 05ZYH33 genome annotation as shown in Figure 4, which included many central biological functions such as metabolism, transcription, translation. To confirm the microarray data, 11 genes (Figure 5) were measured by quantitative RT-PCR. A strong positive correlation (r^2^ = 0.98) between the data obtained by microarray and quantitative RT-PCR suggested the reliability of the microarray data.

To more thoroughly understand the effect of licochalcone A on *S.suis*, we tried to analyze the gene expression profile in the aspect of bacteria cell cycle control. As we know, Bacterial cells, like their eukaryotic counterparts, have a complex subcellular organization required to regulate and coordinate the cell cycle processes. Changes in growth rate must be accompanied by changes in the cell cycle to ensure that cell division stays coordinated with mass doubling, chromosome replication and chromosome segregation [21]. In the presence of licochalcone A, a distinguished change in *S.suis* gene expression profile was the up-regulated expression of ribosomal proteins. Among 54 total genes encoding ribosomal proteins, 23 (42.6%) genes (Table 2) were significantly up-regulated the expression. Besides, genes encoding DNA-directed RNA polymerase were up-regulated and the genes encoding transcriptional regulator were down-regulated. So, the level of gene transcription might be accelerated. These results indicated that *S.suis* might be preparing for the mass doubling. However, we found the genes responsible for DNA replication and cell division did not change or were down-regulated the expression.

As we know, chromosome replication is coordinated with cell growth to ensure that: each origin, replication initiates once and only once per division cycle; at least one round of replication is completed and the nucleoids have segregated before the completion of cell division; and there are sufficient nutrients to support these processes. Nutrient availability is a key determinant for replication initiation [22]. The production of the initiation protein DnaA and other essential components of the replication machinery is proportional to carbon availability and growth rate. Amino acid starvation directly inhibits replication initiation through the production of guanosine tetraphosphate and guanosine pentaphosphate [23]. In the presence of licochalcone A, *S.suis* genes responsible for amino acid transport and metabolism were up-regulated. Among of 29 genes encoding ABC-type amino acid transport system, 11 genes (37.9%) were significantly up-regulated and no gene was significantly down-regulated. Besides, genes responsible for the anabolism of amino acid were up-regulated. For example, gene 05SSU0252 encoding glutamate dehydrogenase and gene 05SSU0791 encoding carbamoylphosphate synthase, were up-regulated more than two fold. However, genes responsible for the catabolism of amino acid were down-regulated. For example, gene 05SSU1709 encoding cysteine aminopeptidase C and gene 05SSU0345 encoding amidotransferase were down-regulated more than two fold. Taken together, we supposed that *S.suis* might be in the status of amino acid starvation and initiation of the replication was inhibited after treatment of licochalcone A.

Division is initiated near the end of chromosome segregation by the formation of a cytokinetic ring at the nascent division site. The tubulin-like protein FtsZ serves as the foundation for assembly of this ring and is required for recruitment of the division machinery. After treated by licochalcone A, as shown in Table 2, *S.suis* cell division related genes encoded by 05SSU0417 (gpsB) and 05SSU0479 are significantly decreased expression two fold, and no gene was significantly up-regulated. Besides, among of 26 cell division related genes based on COG categories, 17 genes down-regulated the expression, 6 genes up-regulated their expression (data not shown).

## Discussion

Licochalcone A is a retrochalcone isolated from the roots and rhizomes of *Glycyrrhiza inflate*. It is active against a wide range of Gram positive organisms but not against Gram negative bacteria and eukaryotes [24]. In this study, our results demonstrated that licochalcone A is active against *S.suis* with MICs of 4 µg/ml for *S.suis* serotype 2 strains and 8 µg/ml for *S.suis* serotype 7 strains. Besides, our results also demonstated that licochalcone A could change some properties of *S.suis* such as biofilm formation and suilysin secretion. Thus, licochalcone A might be a useful compound for the development of prevention and therapy agent for *S.suis* infection.

Antimicrobial agents are often categorized according to their principal mechanism of action. Mechanisms include interference with cell wall synthesis (e.g., β-lactams and glycopeptide agents), inhibition of protein synthesis (macrolides and tetracyclines), interference with nucleic acid synthesis (fluoroquinolones and rifampin), inhibition of a metabolic pathway (trimethoprim-sulfamethoxazole), and disruption of bacterial membrane structure (polymyxins and daptomycin) [25]. The treatment of bacterial infections is increasingly complicated by the ability of bacteria to develop resistance to antimicrobial agents. Licochalcone A had been proved to have potent activity against some Gram positive bacteria, though its antimicrobial mechanism was not well elucidated. One possible mechanism is that licochalcone A could inhibit the bacterial respiratory electron transport chain at the site between Coenzyme Q and cytochrome c [26].

In this study, we tried to investigate the antimicrobial mechanism of licochalcone A in the aspect of bacterial cell cycle control. How organisms adjust their cell cycle dynamics to compensate for changes in nutritional conditions is an important outstanding question in bacterial physiology. Nutrient availability and metabolic status are coordinated with cell growth, chromosome replication and cell division. The gene expression profile of *S.suis* treated by licochalcone A showed that *S.suis* genes responsible for amino acid transport and anabolism of amino acid were up-regulated significantly and genes for catabolism of amino acid were down-regulated. These results indicated that *S.suis* cells might be in the status of amino acid starvation, while amino acid starvation directly inhibits replication initiation through the production of guanosine tetraphosphate and guanosine pentaphosphate. On the other hand, *S.suis* genes for cell division were also been down-regulated. Taken together, we supposed that licochalcone A might inhibit the growth of *S.suis* by controlling the replication initiation and cell division through the amino acid metabolism.

## Conclusions

In this study, we tried to investigate the effect of licochalcone A on growth and properties of *Streptococcus suis*, an important emerging worldwide pig pathogen and zoonotic agent with rapid evolution of virulence and drug resistance. Our results demonstrated that licochalcone A could effectively inhibit the growth, biofilm formation and suilysin secretion of *S.suis*. Besides, we put forward a hypothesis to elucidate the antimicrobial mechanism of licochalcone A in the aspect of bacterial cell cycle control. Namely, licochalcone A might inhibit the growth of S.suis by controlling the replication initiation and cell division through amino acid metabolism. Our results demonstrated that licochalcone A might be a useful compound for the development of prevention and therapy agent for *S.suis* infection.

## Materials and Methods

### Ethics statement

All the experiments in the paper were conducted under the supervision of the Institutional Review Board of the Academy of Military Medical Sciences. All the bacterial isolates were isolated previously and kindly provided by the hospital, Institutes or Academy. No samples were collected from patients directly in this study and therefore the study was exempt from obtaining informed consent. All relevant ethical safeguards have been met in the experiments.

### *Streptococcus suis* and culture conditions

*S.suis* strain 05ZYH33 (previously isolated from an STSS patient with *S.suis* infection) and other *S.suis* strains (listed in Table 1) were used in this study. The microorganism was maintained on Columbia blood agar (BioMerieux) supplemented with 5% sheep blood, and the bacteria were cultured in Todd-Hewitt broth (THB) (Becton, Dickinson and Co.) at 37°C.

### Licochalcone A

Licochalcone A was purchased from Sigma-Aldrich, Inc. and prepared in 99% ethanol at a final concentration of 10 mg/ml and stored at 4°C protected from light at least 24 h to allow sterilization.

### Growth Curves

*S.suis* strain 05ZYH33 was inoculated into THB medium and grown for 12 h at 37°C. And then aliquots of 50 µl of the cultures were inoculated into 5 ml fresh THB medium containing licochalcone A at 0, 0.5, 1.0, 2.0, 4.0 µg/ml in test tubes. Bacteria were further cultured at 37°C and cell growth was monitored spectrophotometrically (OD600 nm at 1 h intervals). 50 µl samples of each culture were collected at 1 h intervals after the addition of Licochalcone A and plated onto THB agar to accurately determine the viable bacteria.

### Determination of minimal inhibitory concentration (MIC)

MICs of different *S.suis* strains were estimated as previously described [24]. Briefly, *S.suis* strain was inoculated into THB medium and grown to mid-log phase (OD600 nm 0.8) at 37°C, aliquots of 50 µl culture of bacteria were inoculated into 5 ml fresh THB medium containing licochalcone A at 0, 0.5, 1.0, 2.0, 4.0 µg/ml in test tubes (For *S.suis* serotype 7, the tested concentration of licochalcone A was up to 8.0 µg/ml) and cultured further for 24 h. The MIC was defined as the lowest concentration at which no visible growth. All assays were performed in triplicate and three independent experiments were carried out.

### Effect of licochalcone A on biofilm formation

The effect of licochalcone A on *S.suis* biofilm formation was assessed by a modification of the methods originally described [14], [27]. *S.suis* strain 05ZYH33 was cultured in broth containing (w/v) 0.5% glucose, 2% peptone, 0.3% K_2_HPO_4_, 0.2% KH_2_PO_4_, 0.01% MgSO_4_·7H_2_O, 0.002% MnSO_4_·6H_2_O, and 0.5% NaCl. Human fibrinogen was also added at concentration of 5 mg/ml to induce biofilm formation. An overnight culture of *S.suis* was diluted in fresh culture broth to obtain an optical density at 600 nm (OD600) of 0.2. Samples (100 µl) were added to the 96-well polystyrene tissue culture plate containing 100 µl of culture medium. Licochalcone A was tested at final concentrations of 2, 3, and 4 µg/ml. After incubation for 18 h or 24 h at 37°C, medium and free-floating bacteria were removed by aspiration and the wells were washed three times with 50 mM phosphate-buffered saline (pH 7.2, PBS). The biofilms were stained with 0.04% crystal violet (100 µl) for 10 min. The wells were washed three times with PBS to remove unbound crystal violet dye and dried for 2 h at 37°C. After adding 100 µl 95% (v/v) ethanol to each well, the plate was shaken for 10 min to release the stain from the biofilms and recorded the absorbance at 550 nm (OD550). All biofilm assays were run in triplicate and the means ± standard deviations of two independent experiments were calculated.

### Titration of hemolytic activity

The suilysin content in *S.suis* culture supernatant was evaluated by titration of its hemolytic activity as previous described with some modifications [28], [29]. Briefly, serial twofold dilutions of test samples were prepared in polystyrene deep-well titer plates with PBS as the diluent. Subsequently, an equal amount of human red blood cells (RBC) (final concentration of RBS: 2%) washed twice in the solution mentioned above, was added to each well. Following incubation for 1 h at 37°C, the mixtures were sedimented by centrifugation (1500 g for 10 min), supernatants were transferred to polystyrene microplates and measured at 540 nm with a microELISA reader.

### *S.suis* whole-genome microarray experiments

*S.suis* strain 05ZYH33 were grown in THB with subinhibitory concentrations (0.25 µg/ml) of LicA to the postexponential growth phase, other aliquots of *S.suis* strain 05ZYH33 without LicA cultured in the same condition were used as control. Immediately before harvesting, 1 volume of bacterial culture was mixed with 2 volume of RNAprotect Bacteria Reageat (Qiagen) by vortexing for 5 s and incubated for 5 min at room temperature to minimize RNA degradition. Then, bacteria were harvested by centrifugation (5000 g for 5 min at 4°C) and total bacterial RNA was extracted by using the MasterPure™ RNA Purification kit (Epicenter). Total RNA was isolated from four replicate cultures at each condition. Microarray Experiments were performed as previously described [30], [31]. Briefly, Total RNA was used to synthesize cDNA in the presence of aminoallyl-dUTP and random hexamer primers. The aminoally-modified cDNA was then labeled with Cy5 or Cy3 dye. Accordingly, four dual-fluorescence-labeled cDNA probes were prepared to hybridize with four slides, respectively. Pairwise comparisons were made using dye swaps to avoid labeling bias. And, the image signals on the slides were captured by using a GenePix personal 4100A microarray scanner. The scan images were processed and data were further analyzed by using GenePix Pro 4.1 software combined with Microsoft Excel software. Spots were analyzed by adaptive quantitation, and the local background was subsequently substracted. Spots with background-corrected signal intensity (median) in both channels of less than twofold of background intensity (median) were rejected from further analysis. Data normalization was performed on the remaining spots by total intensity normalization methods. The normalized log2 ratio of test/reference signal for each spot was recorded. Significant changes in gene expression were identified using SAM software. After SAM analysis, only genes with at least 2-fold changes in expression were collected for further analysis. The microarray data (GSE46666) had been deposited in Gene Expression Omnibus (GEO).

### Real-time quantitative PCR analysis

Gene-specific primers (Listed in Table 3) were designed to produce an amplicon of 100–150 bp for each gene tested. The contaminating DNA in RNA samples was removed by using Amibion's DNA-free Kit (Applied Biosystems, Foster City, CA) cDNAs were generated by random hexamer primers. Using three independent cultures and RNA preparations, real-time RT-PCR was performed in triplicate using the Stepone Plus system together with the SYBR Green master mix. On the basis of the standard curves of 16S rRNA expression, the relative mRNA level was determined by calculating the threshold cycle (ΔCt) of each gene using the classic ΔCt method. Negative controls were performed by using cDNA generated without reverse transcriptase as templates. Reactions containing primer pairs without template were also included as blank controls. The 16S rRNA gene was used as an internal control to normalize all the other genes.

**Table 3:** Genes and oligonucleotides used in validation of DNA microarray data.

Gene	Forward Primer	Reverse Primer
**SSU05_0074**	ACGGTGGTGGTGAAGGTAAAGC	TGGTTGCGACGACGAACGATAAG
**SSU05_0075**	AGGACAACGAACACGGTAAC	CAAATGCTCATCGACGAAAGG
**SSU05_0076**	AGCAGACGCAATCGCAATC	GCTTCGCTGACTACCAAGTTAG
**SSU05_0083**	GGCTTCCGTCTTCGTGAG	AAGTGAAACTGTAACCAATTTGTC
**SSU05_0085**	TTGCCTGCTGGTGTTGAG	GTTTGGACGGTGAAGAGTTAC
**SSU05_0087**	ACAATCCCTCACGAAGTTC	AAGTCACATCTGCGATACC
**SSU05_0089**	GTGGTACATCATCAGGTAACG	GGAAGACGACGGAACAATG
**SSU05_0279**	GCTACTCTGTTGACGGTGGTATG	AGTAACGCCAGCACAAGTGATAG
**SSU05_0280**	TGTCACCTGTTATTGCCGTCTTG	TTCTGCGTCTTCCGTATGAATAGC
**SSU05_0479**	AGTGCTGGCGTCAAAGATGG	TGAAGAAGGTTGGCAGGTAATCC
**SSU05_1759**	ACTATACGAAGGCTGAACCAATC	CTACGAGCATAGAGCACTAAGG
