# Correction: Effect of Licochalcone A on Growth and Properties of *Streptococcus suis*

**DOI:** 10.1371/annotation/59a7568c-915d-4d9c-b912-f93af72acdcb

**Published:** 2013-07-29

**Authors:** Huaijie Hao, Wenjia Hui, Peng Liu, Qingyu Lv, Xiaotao Zeng, Hua jiang, Yanzi Wang, Xin Zheng, Yuling Zheng, Jianchun Li, Xuyu Zhou, Yongqiang Jiang

The author affiliations are not in the correct order. Affiliation 1 and affiliation 2 should be switched. The full, correct list of authors and affiliations can be found below.

Huaijie Hao^1,2☯^

Wenjia Hui^2,3☯^

Peng Liu^2^

Qingyu Lv^2^

Xiaotao Zeng^2^

Hua Jiang^2^

Yanzi Wang^2,4^

Xin Zheng^2,5^

Yuling Zheng^2^

Jianchun Li^3*^

Xuyu Zhou^1*^

Yongqiang Jiang^2*^

 1 CAS Key Laboratory of Pathogenic Microbiology and Immunology, Institute

 of Microbiology, Chinese Academy of Sciences, Beijing, China

 2 State Key Laboratory of Pathogen and Biosecurity, Institute of

 Microbiology and Epidemiology, Academy of Military Medical Sciences,

 Beijing, China

 3 Department of Traditional Chinese Medicine Pharmacology, Shenyang

 Pharmaceutical University, Shenyang, China

 4 Department of Pharmacy, Jiangsu Provincial Xuzhou Pharmaceutical

 Vocational College, Xuzhou, China

 5 Department of Pharmacology, Shenyang Pharmaceutical University,

 Shenyang, China

* E-mail: jiangyq@nic.bmi.ac.cn (YJ); zhouxy@im.ac.cn (X. Zhou); lijianchun0317@sina.com.cn (JL)

☯ These two authors contributed equally to this work.

